# Circulating Exosomes from Mice with LPS-Induced Bone Loss Inhibit Osteoblast Differentiation

**DOI:** 10.1007/s00223-022-00977-x

**Published:** 2022-04-18

**Authors:** Yixuan Wang, Lijun Zhang, Ke Wang, Hua Zhou, Gaozhi Li, Liqun Xu, Zebing Hu, Xinsheng Cao, Fei Shi, Shu Zhang

**Affiliations:** 1 The 940Th Hospital of Joint Logistics Support Force of Chinese People’s Liberation Army, Lanzhou, 730050 Gansu China; 2 The Key Laboratory of Aerospace Medicine, Ministry of Education, Air Force Medical University, Xi’an, 710032 Shaanxi China

**Keywords:** Osteoporosis, Exosomes, Osteoblast

## Abstract

Osteoimmunology focuses on the intermodulation between bone and the immune system. Lipopolysaccharide (LPS)-induced bone loss models are commonly used to investigate the interface between inflammation and osteoporosis. Circulating exosomes can regulate physiological and pathological processes through exosomal microRNAs and proteins. In this study, we observed reduced osteoblast number and bone formation in LPS-induced bone loss mice (LPS mice). Levels of circulating exosomes were increased by ~ twofold in LPS mice, and the expression of exosomal miRNAs was significantly changed. miRNAs (miRNA-125b-5p, miRNA-132-3p, and miRNA-214-3p) that were reported to inhibit osteoblast activity were significantly increased in the serum exosomes and bone tissues of LPS mice. Additionally, LPS-induced increases in exosomes significantly inhibited the osteogenic differentiation of MC3T3-E1 cells.

**Supplementary Information:**

The online version contains supplementary material available at 10.1007/s00223-022-00977-x.

## Introduction

Bone tissue undergoes frequent reconstruction to maintain the stability of the internal environment. The balance between bone resorption and bone formation is crucial for maintaining bone mass and systemic mineral homeostasis [1, 2]. Increased bone resorption and reduced bone formation, which are modulated by hormone fluctuation, mechanical unloading and inflammation, often lead to bone loss and even osteoporosis [3, 4]. Since the concept of osteoimmunology was put forward in 2000, various studies have confirmed that bone tissue and the immune system are closely related and interact with each other [5–7]. Inflammatory factors can induce bone loss, especially chronic and systemic inflammation, which cause diseases, such as rheumatoid arthritis (RA), periodontal disease, osteoarthritis, multiple myeloma, and metastatic bone tumors [8, 9].

Lipopolysaccharide (LPS) is an important component of the outer membranes of Gram-negative bacteria that induce the immune response in humans. Many factors are involved in the process of LPS-induced bone loss, including the local host response, production of prostaglandins and cytokines, and recruitment of inflammatory cells [10, 11]. Recent studies have indicated that the bone loss caused by LPS is not only due to osteoclast activation, but also the inhibition of osteoblast function [12–14]. LPS promotes osteoblast apoptosis and inhibits osteoblast differentiation via activation of the JNK pathway in MC3T3-E1 cells [13].

Exosomes are single-membrane secretory organelles with diameters of 30–200 nm (average ~ 100 nm) that contain selected proteins, lipids, nucleic acids, and glycoconjugates. Exosomes exhibit a variety of activities, including remodeling the extracellular matrix and transmitting signals and molecules to other cells. This exosome-mediated intercellular vesicle transport pathway is involved in human physiological and pathological processes, such as tissue homeostasis, immunity, and cancer [15, 16]. Exosomes also participate in bone remodeling and bone diseases, such as osteoporosis, osteoarthritis, and bone fractures [17, 18]. MicroRNAs (miRNAs) are small endogenous noncoding RNA molecules containing approximately 22 nucleotides that inhibit target gene expression. miRNAs packaged into exosomes can be transported through the intercellular matrix or even into the peripheral blood. Exosomal miRNAs that regulate osteoblast and osteoclast functions have become a focus of research due to their pivotal role in gene expression regulation [15].

In this study, we established an LPS-induced bone loss mouse (LPS mouse) model to research the number of osteoclasts and osteoblasts, and we observed osteoblast activity in vivo through calcein double-labeling experiment and the expression of osteogenic genes. Meanwhile, to investigate the characteristics of exosomes of LPS mice and to study the function of circulating exosomes on osteoblasts, exosomes from Con and LPS mice were isolated and cocultured with MC3T3-E1 cells. The results showed that circulating exosomes from mice with LPS-induced bone loss could inhibit osteoblast differentiation.

## Materials and Methods

### LPS-Induced Bone Loss Model

LPS-induced bone loss is one of the models of osteoporosis caused by inflammation. Male C57BL/6J mice aged 8 weeks were individually maintained under standard conditions (12-h light, 12-h dark cycle, 21 °C controlled temperature) and randomly divided into Con and LPS groups. Mice in the LPS group were intraperitoneally injected with 5 mg/kg LPS (Sigma-Aldrich, USA) on the 1st and 4th day and then anesthetized on the 9th day after the first injection. Bilateral femurs and tibiae were collected and processed for analysis. These study procedures were approved by the Committees of Animal Ethics and Experimental Safety of Air Force Medical University.

### MicroCT Analysis

After fixation in 4% paraformaldehyde for 24 h, each mouse femur was scanned using a microCT scanner (Siemens, Germany) at energies of 80 kV and 500 mA. Femurs were scanned over a total angle of 360° at incremental angles of 0.5°. The scanning time was 800 ms/frame with a resolution of 10.44 μm. A region of interest (ROI) was chosen to represent the microstructure of the femur. The ROI was 15 μm above the proximal epiphyseal growth plate and selected as a 2.5 × 2.5 × 3 mm^3^ cube. The parameters, including BMD, BV/TV, Tb.Th, Tb.N, Tb.Sp and TbPF, were analyzed using COBRA software for microCT. These data were collected for blinded analyses.

### Histology

Collected femurs were fixed in 4% paraformaldehyde, decalcified in 10% ethylenediaminetetraacetic acid (Beyotime Biotechnology, Shanghai, China) and embedded in paraffin. For histological analysis, bone sections were stained with TRAP according to the manufacturer’s protocol (Sigma-Aldrich, USA). For immunohistochemistry, the sections were dewaxed in water, immersed in 5% goat serum, and then incubated overnight at 4 °C with Bglap (1:500; Abcam, USA) primary antibody. Subsequently, diaminobenzidine and hematoxylin were used to assess immunoreactivity. After hard tissue embedding and sectioning, an FV1000 confocal microscope (Olympus, Japan) was used for observation and imaging and hematoxylin was used to detect immunoreactivity. To evaluate the dynamic indexes of bone formation, mice were subcutaneously injected with calcein (Sigma, USA, 8 mg/kg) on the 10th and 3rd day before euthanasia. After embedding and sectioning of hard tissue, an FV1000 confocal microscope (Olympus, Japan) was used for observation and recording. Bone dynamic histomorphometric analysis of the MAR was performed to assess calcium deposition.

### qRT-PCR Analysis

Total RNA was extracted from cells or bone tissues using RNAiso Plus (TaKaRa, Japan), and the RNA in exosomes was extracted using the exoRNeasy Serum/Plasma Kit (Qiagen, USA). cDNA was synthesized using the PrimeScript^®^ RT Master Mix reagent kit (TaKaRa, Japan). The quality and concentration of the total RNA were assessed by measuring absorbance at 260 and 280 nm using a NanoDrop 1000 Spectrophotometer (Thermo Scientific, USA). The PrimeScript^®^ RT Master Mix reagent Kit (TaKaRa, Japan) and the Mir-X miRNA First-Strand Synthesis Kit (TaKaRa, Japan) were used to synthesize cDNA for mRNA and miRNA quantification. Subsequent real-time PCR detection was performed using SYBR^®^ Premix Ex Taq™ II (TaKaRa, Japan) and a CFX96 real-time PCR detection system (BIO-RAD, USA). GAPDH was used as a reference gene, and U6 was used as an endogenous control for normalization. The primers used for real-time PCR are listed in Supplementary Table 1.

### Western Blotting Analysis

Proteins in femurs, exosomes, or MC3T3-E1 cells were collected in RIPA buffer (Thermo Scientific, USA). The same amount of protein samples was loaded onto NuPage Bis–Tris polyacrylamide gels (Invitrogen, USA) and then proteins were transferred onto polyvinylidene difluoride membranes. After blocking in milk (5% w/v) for 4 h at room temperature, the membranes were subsequently incubated overnight at 4 °C with primary antibodies against GAPDH (1:5000; Proteintech, USA), Runx2 (1:1000; Cell Signaling Technology, USA), Bglap (1:500; Abcam, USA), Col1a1 (1:1000; Abcam, US), CD81 (1:1000; Abcam, USA), and TSG101 (1:1000; Abcam, USA). Next, the membranes were incubated with a peroxidase-conjugated secondary antibody (1:5000; Jackson, USA), and the signals were visualized using SuperSignal West substrate (Thermo Fisher Scientific, USA). The density was measured and analyzed using ImageJ software.

### Serum Exosome Preparation

Serum samples (250 μl) from each mouse were collected. Exosomes were isolated by differential centrifugation. Serum samples were centrifuged at 300×*g* for 10 min, 820×*g* for 15 min, and 10,000×*g* for 5 min at 4 °C. Then, sera were pre-filtered to exclude particles larger than 0.8 μm. The supernatants were finally centrifuged at 100,000×*g* for 2 h at 4 °C, washed in PBS, and ultracentrifuged again.

For RNA examination, sera were pre-filtered to exclude particles larger than 0.8 μm. Then, according to the manufacturer’s instructions of the exoRNeasy Serum/Plasma Kit (Qiagen, USA), sera were incubated with XBP buffer. Mixtures were added to the exoEasy spin column and centrifuged at 500×*g* for 1 min. After adding 3.5 ml XBP buffer and spinning at 5000×*g* for 5 min, the spin columns were transferred to collection tubes. Then, 700 μl QIAzol was used to collect the lysate, which was further centrifuged at 1500×*g* for 5 min at 4 °C to collect exosomes.

### Transmission Electron Microscopy

Exosomes were resuspended in 100 μl phosphate buffer. After fixation in 2% paraformaldehyde, exosomes were dropped onto a formvar-carbon-coated grid and left to dry at room temperature. After fixation in 2% glutaraldehyde for 5 min, exosomes were stained with 4% uranyl acetate. The grid was dried at room temperature for 10 min and visualized on a Tecnai G2 F20 transmission electron microscope (FEI, USA) at 120 kV.

### Nanoparticle Tracking Analysis

Exosomes were resuspended in 100 μl of PBS, and nanoparticle tracking analysis measurements were performed using a NanoSight S300 system (Version 3.2 Build 3.2.16, UK). Exosomes were captured using a sCMOS camera at 25.4 °C, followed by measurement of concentration and particle modal size.

### miRNA Sequencing

Exosomes from five Con or LPS mouse serum samples were examined as one sample, and total RNA from exosomes was subjected to miRNA sequencing. Preparation and sequencing of miRNA libraries were performed by RiboBio (Guangzhou, China). Small RNAs of 18–30 nt were utilized for library preparation. PCR amplification products were sequenced using the Illumina HiSeq 2500 platform.

### FACS of Osteoblast

Bone marrow cells were taken from bilateral femurs of C57BL/6J mice. The ALP population was stained with primary antibodies against (1:50; R&D, USA) and phycoerythrin-conjugated anti-goat IgG secondary antibody (1:200, R&D, USA). The incubated cells were sorted and analyzed by FACS aria II flow cytometry (BD Biosciences, USA). Selected ALP^+^ cells were used for total RNA extraction and then analyzed by real-time PCR.

### Cell Culture

The mouse preosteoblast MC3T3-E1 cell line was purchased from the Cell Bank of the Chinese Academy of Sciences (Shanghai, China). MC3T3-E1 cells were cultured in α-MEM (Gibco) containing 10% FBS (HyClone) and 1% penicillin and streptomycin (HyClone). Cells were maintained under standard cell culture conditions of 5% CO_2_, 95% humidity, and 37 °C. Eight to 12 generation cells were used in the experiments. To examine the function of osteogenic differentiation, MC3T3-E1 cells were induced using osteogenic medium supplemented with 100 nM dexamethasone, 50 μM ascorbic acid, and 10 mM β-glycerophosphate (Sigma).

### Exosome Labeling and Uptake by Cells

To determine whether serum exosomes from LPS mice could be taken up into MC3T3-E1 cells, exosomes were collected and stained with PKH26 (Sigma-Aldrich, USA) for 4 min at room temperature. After adding 2 ml 0.5% BSA to stop labeling, exosomes were collected and resuspended in PBS. Ten micrograms/ml PKH26-labeled exosomes were added to the culture medium of MC3T3-E1 cells and incubated for 12 h. Nuclei were counterstained with DAPI. Exosomes taken up by cells were examined using a confocal microscope (Olympus FV1000, Japan).

### Alkaline Phosphatase Staining

After MC3T3-E1 cells were cultured in osteogenic medium for 7 days, ALP staining was performed using an NBT/BCIP staining kit (Beyotime Biotechnology, China) according to the manufacturer’s protocol. Staining for each group was replicated at least three times, and representative images were recorded using a digital camera.

### Statistical Analysis

All statistical analyses were performed using SPSS 22.0 software. All numerical data are shown as the mean ± SD from at least three or five duplicate experiments. Statistical significance was tested using two-tailed* t* test or one-way ANOVA. A *P* value < 0.05 was considered to be significant.

## Results

### LPS Induces Bone Loss in Mice

**Fig. 1:**
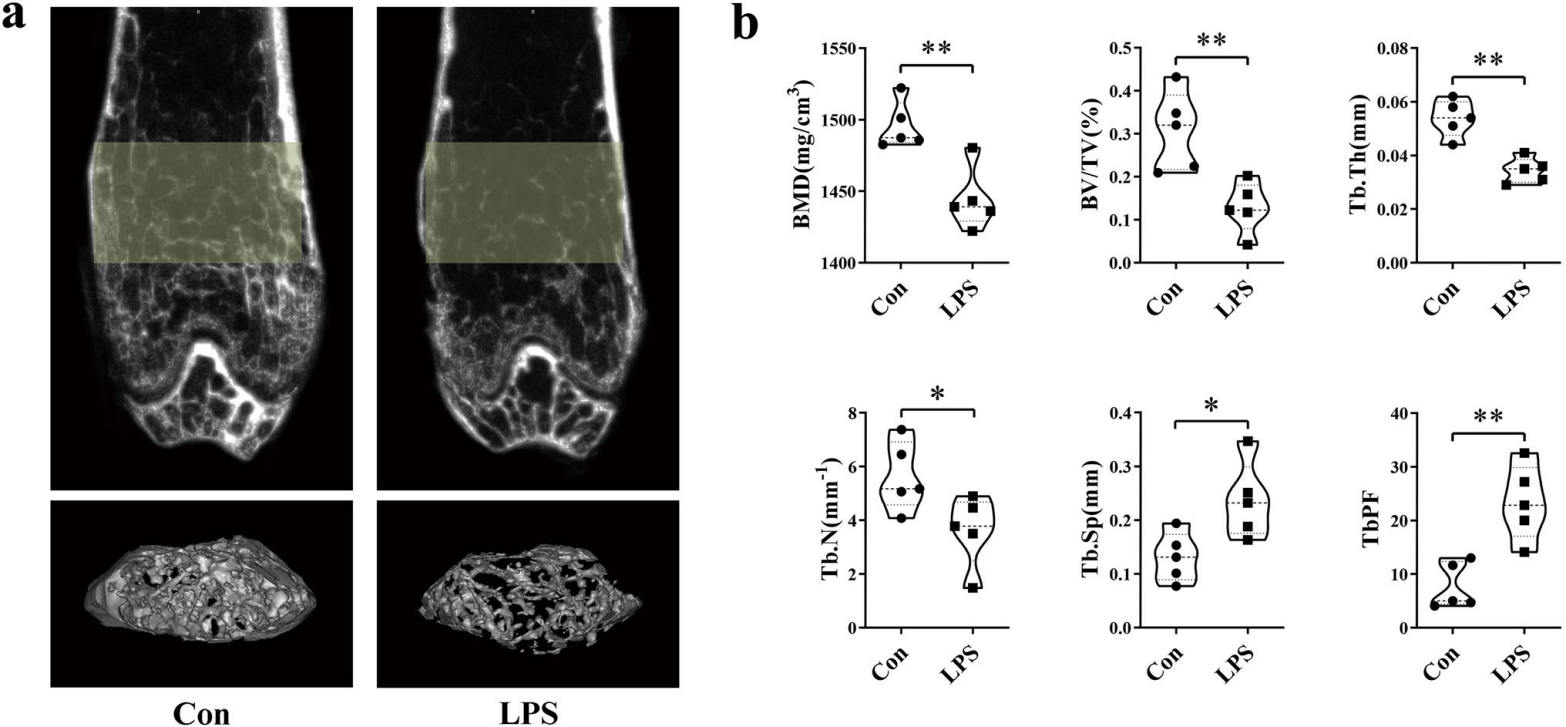
LPS induces bone loss in mice. **a** Representative images were examined by microCT to assess the trabecular architecture of the distal femurs of mice from each group. **b**Three-dimensional microstructure of the ROI region of the distal femurs, including bone mineral density (BMD), relative bone volume (BV/TV), trabecular thickness (Tb.Th), trabecular number (Tb.N), trabecular space (Tb.Sp), and trabecular pattern factor (TbPF) (*N* = 5). *P < 0.05, **P < 0.01 vs. Con

To explore the function of circulating exosomes in osteoporosis, LPS-induced bone loss mice were selected as a model. Mice in the experimental group were injected with LPS twice on the 1st and 4th days. On the 9th day, the femurs in LPS mice presented a typical bone loss phenotype, exhibiting severely damaged trabecular structures and losses in bone mass (Fig. 1a). Three-dimensional architecture parameters also showed corresponding changes with significant decreases in bone mineral density (BMD), relative bone volume (BV/TV), trabecular thickness (Tb.Th), and trabecular number (Tb.N) and remarkable increases in trabecular separation (Tb.Sp) and trabecular pattern factor (TbPF) (Fig. 1b).

### Osteoblast Differentiation is Inhibited in LPS-Induced Bone Loss Mice

**Fig. 2:**
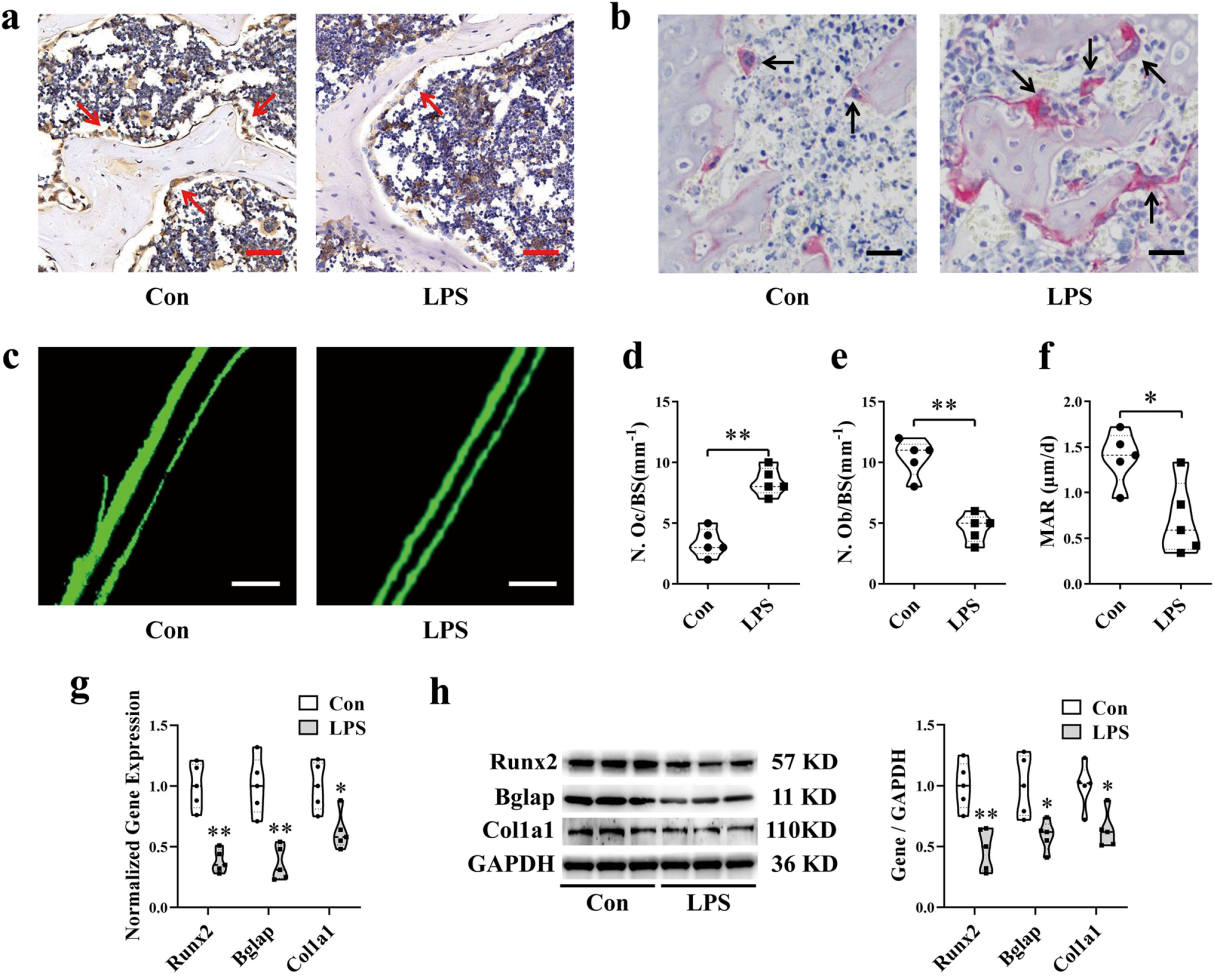
Osteoblast differentiation is inhibited in LPS-induced bone loss mice. **a** Representative images of TRAP staining of the distal femurs of mice in each indicated group. Scale bar, 50 µm. **b** Representative images of Bglap staining of the distal femurs of mice in each indicated group. Scale bar, 50 µm. **c** Representative images of new bone formation assessed by double calcein labeling. Scale bar, 20 µm. **d** Statistics of the number of positive osteoclasts per bone surface of the femur (N.Oc/BS) (*N* = 5). **e** Statistics of the number of positive osteoblasts per bone surface of the femur (N.Ob/BS) (*N* = 5). **f** Statistical analysis of the histological parameter MAR (*N* = 5). **g** qRT-PCR analyses of Runx2, Bglap, and Col1a1 levels in the tibial bone tissue of CON and LPS mice (*N* = 3). **h** Protein levels of Runx2, Bglap, and Col1a1 in the tibial bone tissue of CON and LPS mice (*N* = 3). *P < 0.05, **P < 0.01 vs. Con

To investigate characteristics of osteoclasts and osteoblasts in LPS mice, we examined histomorphometric changes in the distal femurs. TRAP staining showed that osteoclasts were significantly increased in the femurs of LPS-treated mice compared to those of control (Con) mice (Fig. 2a, d). Bglap-positive osteoblasts in the distal femur were decreased in the LPS group (Fig. 2b, e). Meanwhile, we did experiments to research the osteoblasts activity in vivo. Calcein double labeling indicated that bone formation was significantly decreased in LPS mice compared with that in Con mice. Regarding bone histomorphometric parameters, the mineral apposition rate (MAR) showed the same trends (Fig. 2c, f). Furthermore, qRT-PCR and Western blot results showed that expression levels of the osteogenic genes Runx2, Bglap, and Col1a1 were all significantly decreased in the femurs of LPS mice (Fig. 2g, h).

### Circulating Exosome Levels are Increased in LPS Mice

**Fig. 3:**
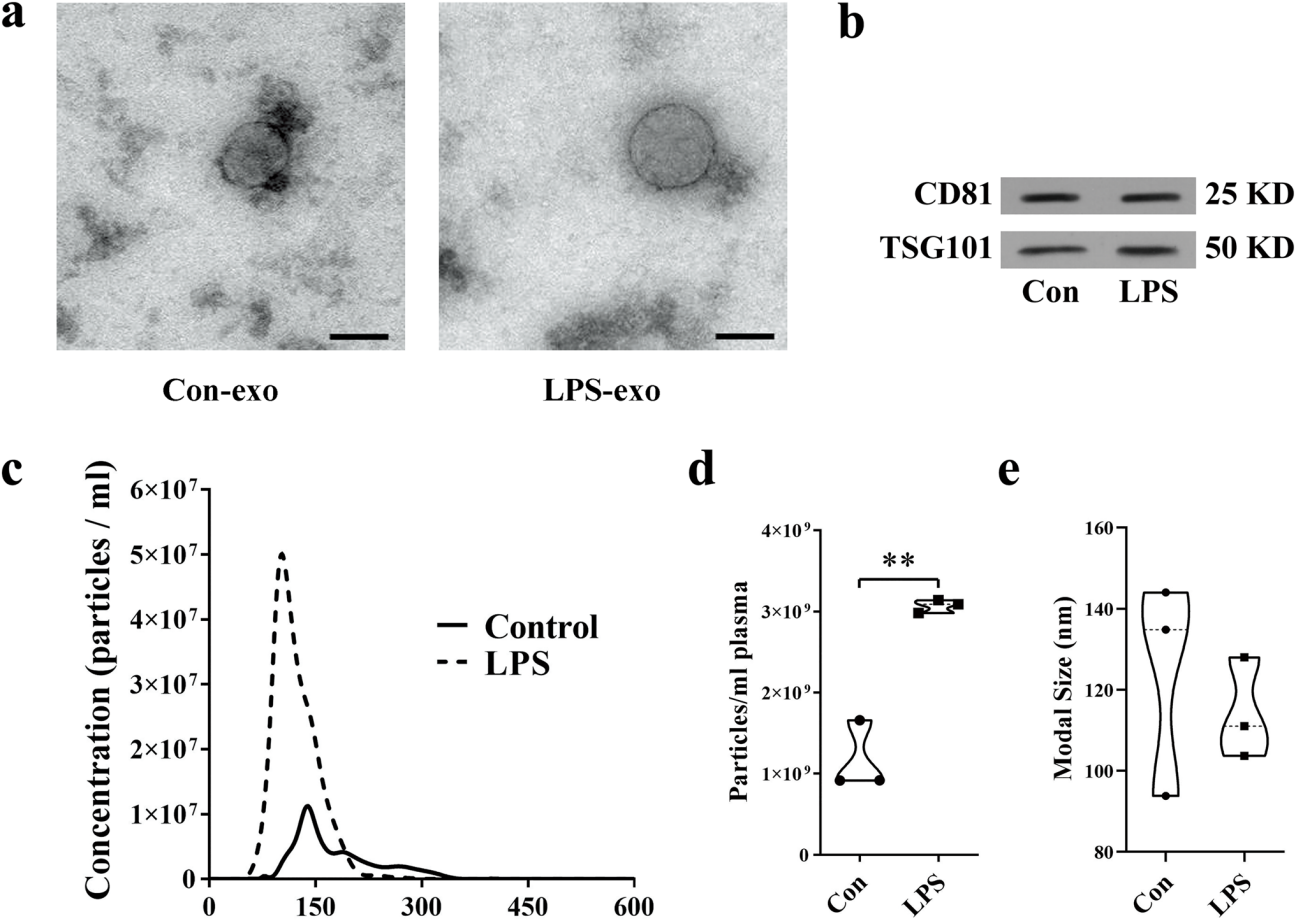
Circulating exosome levels are increased in LPS mice. **a** Representative images of transmission electron microscopy for exosomes isolated from the serum. Scale bar, 100 nm. **b** Protein levels of the exosomal markers CD81 and TSG101 in exosomes (*N* = 3). **c**–**e** Nanoparticle tracking analysis of the average concentration and modal size of exosomes in the serum (*N* = 3). **P < 0.01 vs. Con

To determine whether LPS affects circulating exosome levels, we extracted exosomes from serum and processed them for analysis. We observed that the diameter of serum exosomes was approximately 100 nm using transmission electron microscopy (TEM) (Fig. 3a). Western blot analysis revealed that the exosomal markers CD81 and TSG101 were expressed in exosomes from both Con and LPS mice (Fig. 3b). Using nanoparticle tracking analysis, we found that the number of circulating exosomes isolated from the serum of LPS mice was increased by approximately twofold, while the modal size of the particles did not change significantly (Fig. 3c–e).

### Expression of miRNAs is Significantly Altered in the Exosomes and Femurs of LPS Mice

**Fig. 4:**
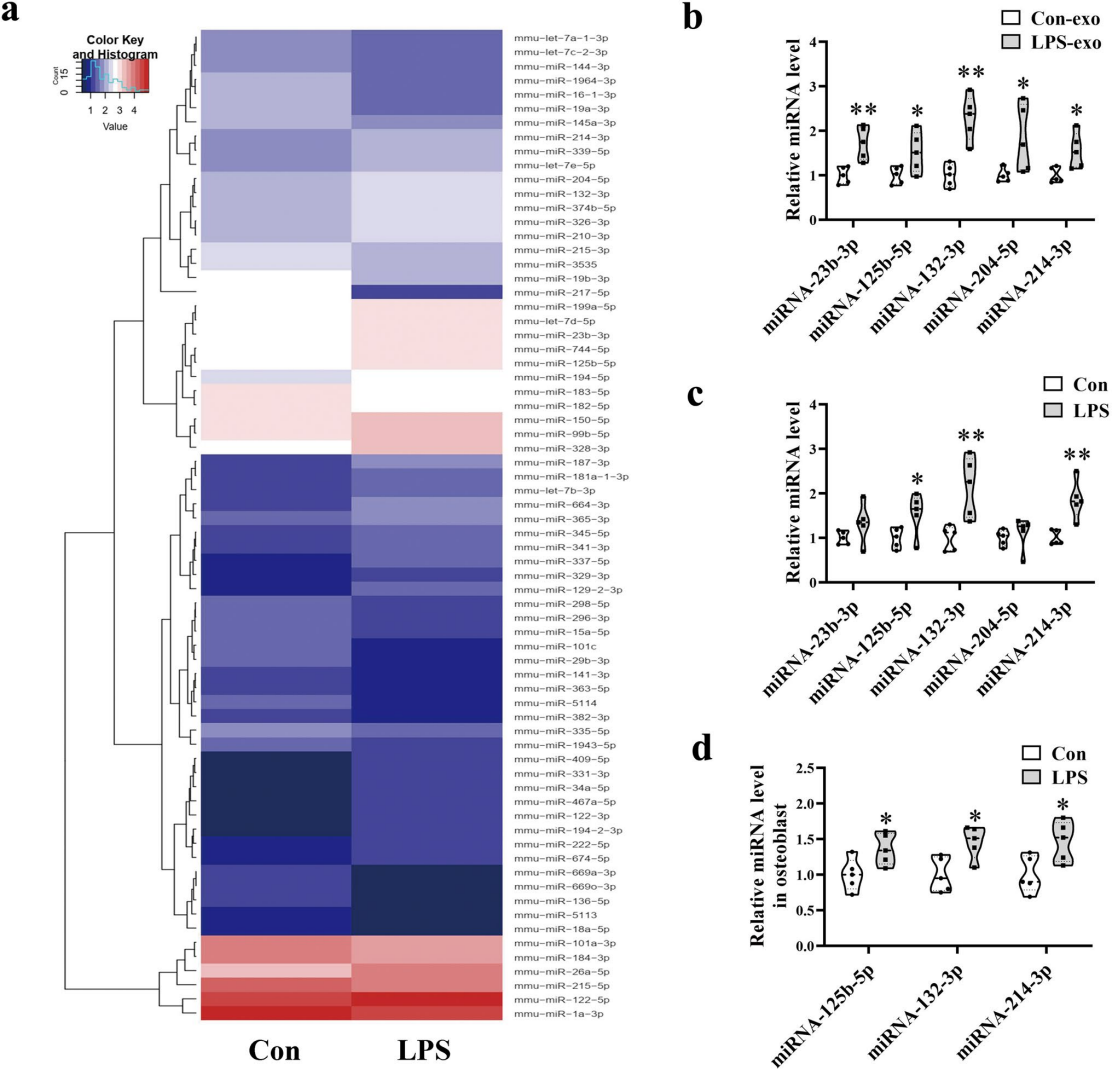
Expression of miRNAs is significantly altered in exosomes and femurs of LPS mice. **a** Heatmap diagram of differential miRNA expression between exosomes derived from exosomes isolated from Con and LPS mouse serum. Mean expression values are shown. **b** qRT-PCR analyses of miR-23b-3p, miR-125b-5p, miR-132-3p, miR-204-5p, and miR-214-3p levels selected from the sequencing data of exosomes in the serum (*N* = 5). **c** qRT-PCR analyses of miR-23b-3p, miR-125b-5p, miR-132-3p, miR-204-5p, and miR-214-3p levels in the tibial bone tissue of Con and LPS mice (*N* = 5). **d** qRT-PCR analyses of miR-125b-5p, miR-132-3p, and miR-214-3p levels in osteoblast isolated from bone marrow of Con and LPS mice (*N* = 5). *P < 0.05, **P < 0.01 vs. Con

To investigate the miRNAs in exosomes, gene expression profiling of Con and LPS mice was analyzed by RNA sequencing. Exosomes from five Con or LPS mouse serum samples were examined as one sample. Expression levels of 70 miRNAs were altered, as shown in the hierarchical clustering analysis heatmap (Fig. 4a). Among them, miRNA-23b-3p, miRNA-125b-5p, miRNA-132-3p, miRNA-204-5p, and miRNA-214-3p, which are reported to be negatively correlated with osteogenic differentiation [19–23], were selected as candidate miRNAs. qRT-PCR analysis revealed that expression levels of these miRNAs were significantly upregulated in exosomes of LPS mice (Fig. 4b). Meanwhile, we assessed the expression of these miRNAs in femurs and found that miRNA-125b-5p, miRNA-132-3p, and miRNA-214-3p were also increased in the LPS group (Fig. 4c). Furthermore, we isolated osteoblast (ALP^+^ cells) from bone marrow cells of CON and LPS mice by FACS (fluorescence-activated cell sorting). Using qRT-PCR to examine miRNA-125b-5p, miRNA-132-3p, and miRNA-214-3p expression, we found that miRNA-125b-5p, miRNA-132-3p, and miRNA-214-3p were increased in osteoblast of LPS mice (Fig. 4d).

### Circulating Exosomes from LPS Mice Inhibit Osteoblast Differentiation in MC3T3 Cells

**Fig. 5:**
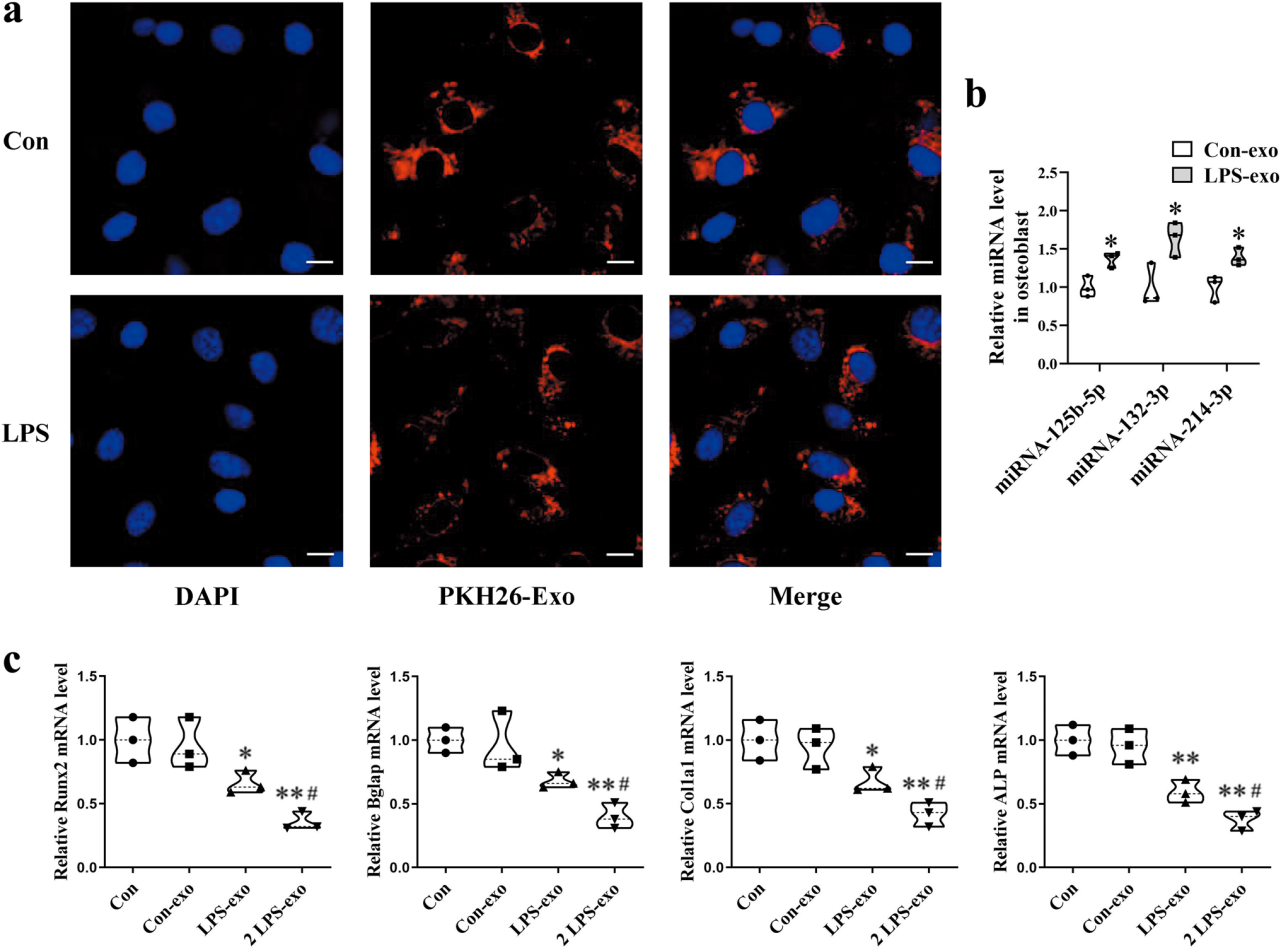
Circulating exosomes from LPS mice inhibit the mRNA levels of osteogenic genes in MC3T3-E1 cells. **a** Confocal microscopy of MC3T3-E1 cells treated with exosomes labeled with PKH26. Scale bar, 25 µm. **b** qRT-PCR analyses of miR-125b-5p, miR-132-3p, and miR-214-3p levels in osteoblast (*N* = 3). **c** qRT-PCR analyses of Runx2, Bglap, Col1a1, and ALP levels in osteoblasts (*N* = 3). *P < 0.05, **P < 0.01 vs. Con-exo and ^**#**^*P* < 0.05 vs. LPS-exo

To investigate the function of circulating exosomes on osteoblasts, exosomes from Con (Con-exo) and LPS (LPS-exo) mice were isolated. MC3T3-E1 cells were cultured with PKH26 fluorescently labeled exosomes from Con and LPS mice. Confocal microscopy results revealed that circulating exosomes could be transferred into osteoblasts, indicating that exosomes play a potential role in regulating osteoblast function (Fig. 5a). MC3T3-E1 cells were cocultured with equal amount of Con-exos and LPS-exos for 48 h. qRT-PCR analysis revealed that expression levels of miRNA-125b-5p, miRNA-132-3p, and miRNA-214-3p were increased in LPS-exo group than Con-exo group (Fig. 5b).

**Fig. 6:**
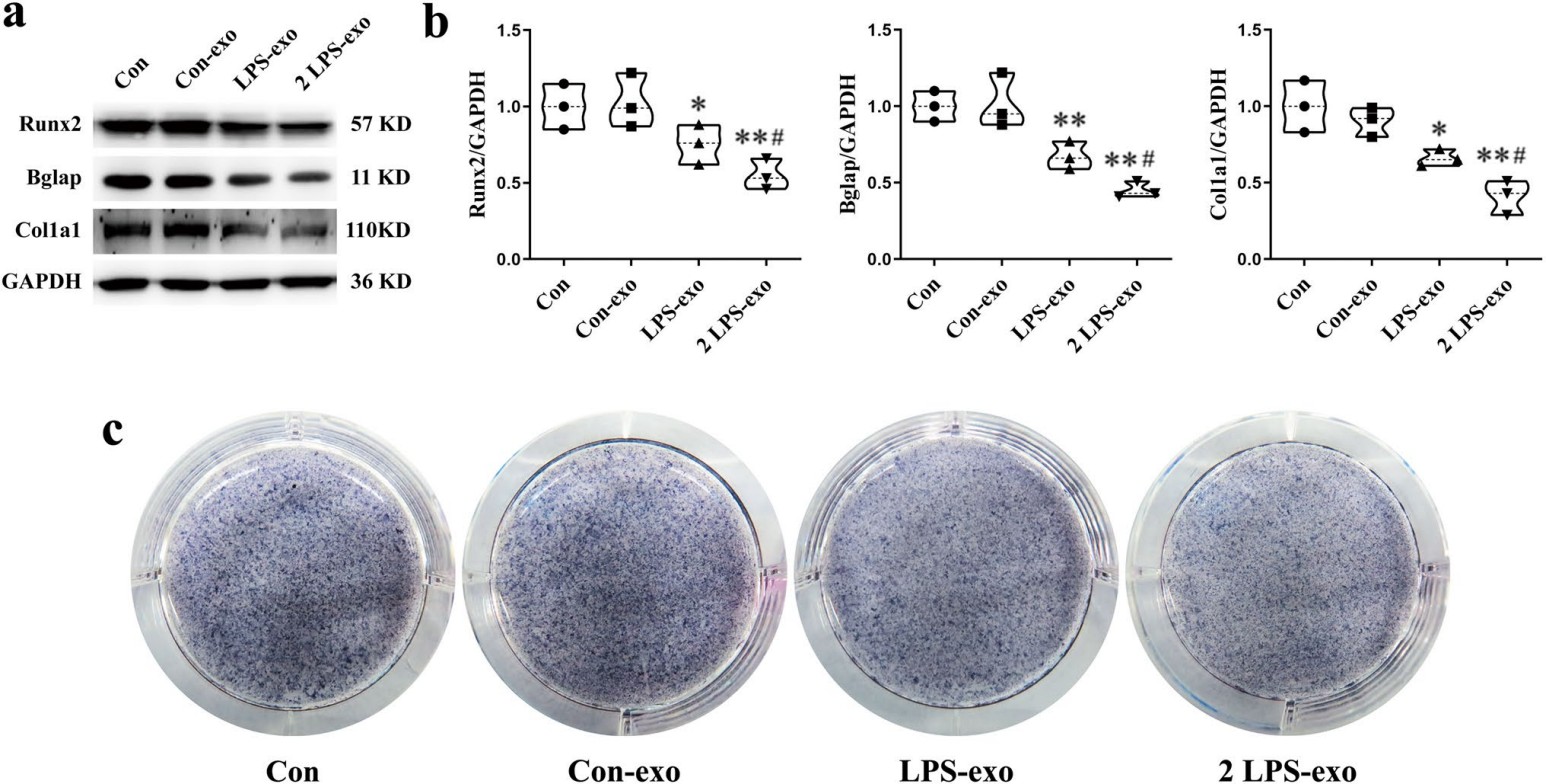
Circulating exosomes from LPS mice inhibit the protein levels of osteogenic genes in MC3T3-E1 cells. **a**, **b** Western blot analysis of Runx2, Bglap, and Col1a1 protein expression in osteoblasts (*N* = 3). *P < 0.05, **P < 0.01 vs. Con-exo and ^#^*P* < 0.05 vs. LPS-exo. **c** Representative images of ALP staining in osteoblasts (*N* = 3)

We next investigated whether LPS-exos and the increase in the number of exosomes inhibit osteoblast differentiation. As we have already identified that the number of circulating exosomes isolated from the serum was increased by ~ twofold in response to LPS injection, MC3T3-E1 cells were cultured with Con-exos, LPS-exos, and twofold LPS-exos. qRT-PCR analysis showed that expression levels of the osteogenic genes Runx2, Bglap, Col1a1, and ALP were significantly downregulated in the LPS-exo and 2 LPS-exo groups compared to the Con-exo group. Furthermore, the twofold LPS-exo group inhibited the mRNA expression of osteogenic genes in osteoblasts more strongly than the LPS group (Fig. 5c). These changes were also examined at the protein level. Protein expression of the osteogenic genes Runx2, Bglap, and Col1a1 was reduced in MC3T3-E1 cells cultured with exosomes isolated from LPS mouse serum, and the effect of twofold LPS exosomes was more significant, as indicated by Western blotting (Fig. 6a, b). ALP staining was also significantly decreased in the LPS-exo and twofold LPS-exo groups compared to the Con group, while this decrease was more obvious in the twofold LPS-exo group (Fig. 6c). These results indicated that exosomes from LPS mouse serum inhibit osteoblast differentiation and that the increase in LPS-derived exosomes further enhances this effect.

## Discussion

Osteoimmunology focuses on the intermodulation between bone and the immune system. Activation of immune pathways can regulate the number and function of bone cells, all of which affect the balance between bone formation and bone resorption in healthy and diseased states [24]. Inflammatory factors can cause osteoporosis, and many types of inflammatory bone loss are due to LPS [25]. LPS-induced bone loss models are commonly used to investigate the interface between inflammation and osteoporosis. Our research demonstrated that mice injected with LPS presented a typical bone loss phenotype. The number of osteoclasts was significantly increased, while the number of osteoblasts and bone formation was decreased in LPS-treated mice.

Exosomes are secreted into the blood by various cell types and regulate both physiological and pathological processes through the circulatory system. LPS can induce systemic or local immune responses, leading to changes in circulating exosomes, which can affect the function of other systems. In a previous study, LPS was used to establish endotoxemia in mice, and inflammatory microRNAs were significantly upregulated in the circulating exosomes of LPS-injected mice. Purified exosomes from mice with endotoxemia that were intravenously injected into normal adult mice led to elevated microglial activation, which indicates that circulating exosomes may act as neuroinflammatory mediators in systemic inflammation [26]. After intratracheal instillation of LPS, microvesicles containing miR-223/142 were dramatically induced in bronchoalveolar lavage fluid and serum, which suppressed macrophage activation and lung inflammation [27]. Another study also used LPS to induce acute respiratory distress syndrome (ARDS). Circulating exosomes from LPS mice trigger endoplasmic reticulum stress in lung tissue and facilitate the development of ARDS [28]. In our study, we used LPS to construct a bone loss mouse model and subsequently purified exosomes from LPS-injected mouse serum. The modal size of the particles did not change significantly, but the number of circulating exosomes isolated from the serum of LPS mice increased by approximately twofold, and expression levels of exosomal miRNAs were significantly altered.

Noncoding RNAs are abundant in exosomes and play critical roles in bone remodeling. Exosomal miRNAs can be transferred from bone cells, including osteoclasts, osteoblasts, and their precursors, to target cells, where they regulate gene expression [15]. Studies on bone loss in RA demonstrated that miRNAs were significantly altered in inflamed synovial tissue between nonarthritic and arthritic mice. MiR-221-3p in exosomes was upregulated by synovial fibroblasts treated with the proinflammatory cytokines and suppressed osteoblast differentiation and mineralization in vitro [29]. MiR-214 levels are significantly increased in serum exosomes from osteoporotic patients and mice, which were associated with reduced bone formation. Osteoclast-derived exosomal miR-214-3p could transfer to osteoblasts to inhibit osteoblast differentiation in vitro [30, 31]. After injection of exosomes secreted by bone marrow mesenchymal stem cells (BMSCs) into ovariectomized osteoporosis rats, miR-150-3p levels were increased in bone tissues and attenuated osteoporosis in vivo [32]. Our study found that miRNA expression was distinct in serum exosomes from LPS-induced bone loss mice. Among them, miRNA-125b-5p, miRNA-132-3p, and miRNA-214-3p were increased in serum exosomes and femurs of LPS mice. These miRNAs were reported to be negatively correlated with osteogenic differentiation. MiRNA-125b-5p is downregulated during the process of periodontal ligament cell (PDLC) differentiation and enhances NF-κB signaling to attenuate osteoblast differentiation [20]. MiRNA-132-3p was increased in bone tissues of unloading osteoporosis rats and inhibited primary rat osteoblast differentiation by targeting Ep300 [21]. Several studies also confirmed that miRNA-214-3p inhibits osteoblast differentiation and mineralization [23, 30, 31, 33]. Furthermore, increases in exosomal miRNA-125b-5p, miRNA-132-3p, and miRNA-214-3p aggravated bone loss in LPS-injected mice.

Previous studies demonstrated that circulating exosomes influence the function of osteoblasts and osteoclasts to regulate bone remodeling. Serum exosomes derived from osteoporosis patients suppress the activation of osteoblasts and trigger the resorption of osteoclasts. Serum exosomes derived from osteopenia patients enhanced both osteoblast function and osteoclast activation, which could lead to a compensatory elevation in bone remodeling [34]. Human monocytes stimulated by LPS release exosomes that contained a wide size distribution of RNA, including RNA in the size of microRNAs. Exosomes can be taken up by hMSCs, and osteogenic differentiation of hMSCs cocultured with exosomes increased [35]. Our study determined that serum exosomes could be absorbed by MC3T3-E1 cells. Expression of the osteogenic genes Runx2, Bglap, Col1a1, and ALP in osteoblasts cocultured with LPS-exos was significantly decreased. Furthermore, with the increase in the number of cocultured LPS-exos, the effect of inhibiting osteoblast differentiation was enhanced. These results indicate that LPS-induced changes in the number of circulating exosomes and the expression of exosomal miRNAs may be responsible for the inhibition of osteoblast activity.

In conclusion, this study revealed that circulating exosomes in LPS-induced bone loss mice were increased, and the expression of exosomal miRNAs was significantly altered. LPS-induced increases in exosomes significantly inhibited osteoblast differentiation. Circulating exosomes might serve as a potent source for aggravating inflammatory bone loss.

## Supplementary Information

Below is the link to the electronic supplementary material.

Supplementary file1 (DOCX 18 kb)
