# Supplementary material for: Circulating Exosomes from Mice with LPS-Induced Bone Loss Inhibit Osteoblast Differentiation
Source: Calcif Tissue Int. 2022 Apr 18;111(2):185–95. doi: 10.1007/s00223-022-00977-x (PMC9300544; doi:10.1007/s00223-022-00977-x)
Supplement: Supplementary file 1 — Supplementary file1 (DOCX 18 kb) [file 223_2022_977_MOESM1_ESM.docx]

**Supplemental information**

**Supplement Circulating exosomes from LPS-induced bone loss mice inhibit osteoblast differentiation.**

Yixuan Wang^1，2^, Lijun Zhang^2^, Ke Wang^2^, Hua Zhou^2^, Gaozhi Li^2^, Liqun Xu^2^, Zebing Hu^2^, Xinsheng Cao^2^, Fei Shi^2^, Shu Zhang^2^

1 The 940th Hospital of Joint Logistics Support Force of Chinese People’s Liberation Army

2 The Key Laboratory of Aerospace Medicine, Ministry of Education, Air Force Medical University, 710032, Xi'an, Shaanxi, China.

* YXW, LJZ and KW contributed equally to this work.

**Supplement Table 1. The sequence of primers and siRNAs.**

| Name | Sequence (5'-3') |
| --- | --- |
| Runx2-F | GAA CCA AGA AGG CAC AGA CAG A |
| Runx2-R | GGC GGG ACA CCT ACT CTC ATA C |
| ALP-F | GCA GTA TGA ATT GAA TCG GAA CAA C |
| ALP-R | ATG GCC TGG TCC ATC TCC AC |
| Bglap-F | GAC CGC CTA CAA ACG CAT CTA |
| Bglap-R | CAG AGA GAG AGG ACA GGG AGG A |
| Col1a1-F | GAC ATG TTC AGC TTT GTG GAC CTC |
| Col1a1-R | GGG ACC CTT AGG CCA TTG TGT A |
| GAPDH-F | TGT CCG TCG TGG ATC TGA |
| GAPDH-R | TTG CTG TTG AAG TCG CAG GAG |
| miR-23b-3p | CAC ATT GCC AGG GAT TAC C |
| miR-125b-5p | CCT GAG ACC CTA ACT TGT GA |
| miR-132-3p | CAG TCT ACA GCC ATG GTC G |
| miR-204-5p | CCC TTT GTC ATC CTA TGC CT |
| miR-214-3p | CAG GCA CAG ACA GGC AGT |
